# Identification of Dynamic Patterns of Personal Positions in a Patient Diagnosed With Borderline Personality Disorder and the Therapist During Change Episodes of the Psychotherapy

**DOI:** 10.3389/fpsyg.2022.716012

**Published:** 2022-08-03

**Authors:** Augusto Mellado, Claudio Martínez, Alemka Tomicic, Mariane Krause

**Affiliations:** ^1^Doctoral Program in Psychotherapy, Department of Psychology, Pontificia Universidad Católica de Chile, Santiago, Chile; ^2^Faculty of Psychology, Universidad Diego Portales, Santiago, Chile; ^3^Department of Psychology, Faculty of Social Sciences, Pontificia Universidad Católica de Chile, Santiago, Chile

**Keywords:** voices and personal positions, change episodes, subjective change, dynamic patterns, process and outcomes

## Abstract

Personal positions and voices of a patient diagnosed with borderline personality disorder (BPD) and the therapist during long-term psychotherapy were studied aiming to find differences in the patterns formed in these aspects of subjectivity according to the level of elaboration of the change episodes achieved by the patient. This case study considered a stage of qualitative analysis where change episodes of the patient were traced through the Change Episodes Model. Later, through the Model of Analysis of Discursive Positioning in Psychotherapy (MAPP), the voices and personal positions of the patient and her therapist were identified in each of the change episodes. In the stage of quantitative analysis, dynamic patterns in the voices and personal positions were established, accounting for hypothetical attractors using the Space State Grid (SSG) technique in each of the three different levels of subjective elaboration that constitute the change episodes. The results established differentiated dynamic patterns in the change episodes, coherent with the patient’s change process, and formation of propositive/reflective specific patterns as the patient evolved in the three different levels of subjective elaboration. The above suggests that a subjective transformation process is displayed, and this is manifested in the different voices and personal positions that emerged as the change episodes evolve. The identified dynamic patterns can be considered nonlinear and emergent subjective exchanges between the patient and the therapist throughout the psychotherapy.

## Introduction

Psychotherapeutic change is often evaluated using indicators of symptoms, self-reports of general psychological well-being, and other measures and instruments. It is also important to consider the change in the subjectivity of patients and its relevance at different key moments of psychotherapy. One of the main ways to access subjectivity is through the discourse of patients and therapists during a therapeutic process. For this, there is the notion of personal positions identified in the discourse (Martínez and Tomicic, 2019), which can be understood as a manifestation of different self states (Bromberg, 1994; Hermans, 2003; Hermans and Dimaggio, 2004). Furthermore, some perspectives of change in psychotherapy increasingly emphasize its self-organizing and emerging character, thus establishing the need to consider studying the reorganization of subjectivity from this perspective.

Finally, observing a change in the subjectivity of a patient in the context of therapeutic relationship can be valuable in a clinical case where, according to the patient’s diagnosis, a process can be observed that starts from an experience of dissociation from the self that can be integrated over time. Then, studying the case of a patient diagnosed with borderline personality disorder (BPD) may provide relevant conclusions in this regard, since different issues such as dissociation and identity diffusion (Laddis et al., 2017; Scalabrini et al., 2017; Luyten et al., 2020) associated with vulnerability of the self have been described in these patients.

There are different paradigmatic approaches to the notion of “self” expressing in psychotherapy through the discourses of therapists and patients (Georgaca, 2001, 2003; Hermans and Dimaggio, 2004; Gonçalves and Guilfoyle, 2006; Dimaggio and Stiles, 2007; Hermans, 2008, 2014; Avdi and Georgaca, 2009; Clegg and Salgado, 2011; Avdi, 2012).

One such approach, coming from a dialogical perspective, points out that the self is composed of several kinds of I-positions or self states (Bromberg, 1996; Hermans, 2004) that find themselves in a constant interaction as individuals engage in different internal and external dialogues. Specifically, a personal position can be understood as a possible state of the self, among multiple other possible states, that depends on a specific background and whose emergence in a given moment will be about, among other aspects, the others (potential audiences), being able to change from one moment to another (Salgado and Hermans, 2009). For example, an individual could speak from a negotiating position in front of a family member and be intransigent in a work meeting, even though the situation may be similar. According to Bromberg (1996), an individual is only weakly aware of the existence of individual self states, because people maintain a healthy illusion of cohesive personal identity.

The “I” can move, as if it were in a space, from one position to another according to changes that can be experienced in different situations and in time, endowing each position with a voice or voices (in a metaphoric way) that can give them expression in the discourse and allow for dialogical relationships among the different positions. Many positions can be occupied by the same individual, and they can even manifest contradictorily, although some positions can organize others at some time (Hermans et al., 1992; Hermans, 2004, 2008, 2014). Furthermore, by including the notion of a polyphonic novel of Bakhtin (Hermans, 2001, 2004), the idea notion of open dialogue in the inner world of individuals in the form of an interpersonal relationship that grants multiple perspectives (Hermans, 2001, 2004) can be established.

From this perspective, the others are presented in a double protagonist position, internal and external, that is constituted during the history of interpersonal relationships and that situates them as characters that form part of the self in an inner scenario, influencing the subjective dynamics (Hermans and Dimaggio, 2004). The “I” can act imaginatively as if it were the others, generate supposed conversations, and interpret them as a position that the “I” can occupy and that can create an alternative point of view of the self and the world. An individual can address another person in an external dialogue and simultaneously hold an internal dialogue with potential audiences, directly related to the subject of discussion. These dialogues can also be established with objects, other living forms or oneself. The dialogue with one’s self also considers that there are private (subjective) experiences that are not necessarily shared with others but are part of the communicational process (Salgado and Hermans, 2009).

Some characteristics of the positions are that they may be transitory or more permanent; they may be more or less sustained and established by institutional traditions; they may differ in their influence over others; they may be more or less imaginary; they may enter the self more frequently than others; they may vary between positive and negative dimensions (pleasant, annoying, and even threatening), and in their degree of alterity (Hermans, 1996).

In psychotherapy, therapists have the possibility of becoming a new position in the subjectivity of a patient. From this perspective, dysfunctional elements of the self can be changed as part of a repertoire of broader organized positions. Functional parts of the self can facilitate the modification of dysfunctional parts and enable their inclusion as components of broader and more adaptive coalitions (Hermans and Dimaggio, 2004).

The self has multiple parts or multiple facets that manifest, stress, and change in psychotherapy (Osatuke et al., 2004; Dimaggio and Stiles, 2007). According to Stiles, a change process involves understanding the self as an active community of interacting agents or voices in which problematic experiences would constitute active voices that have their feelings and motivations (Honos-Webb and Stiles, 1998). He considers a dialogical self immersed in an assimilation process, composed of a set of steps starting from a constrained self, where a symptom may correspond to a voice demanding to be heard, or a manifestation of a rejected voice, to a self made up of a cast of characters or voices that have been accepted in the experience (Honos-Webb and Stiles, 1998; Osatuke et al., 2005).

Specifically, the psychotherapeutic dialogue between two participants involves two dimensions that are expressed simultaneously: one inter-mental (external dialogue) and the other intra-mental (internal dialogue). The first dimension occurs in observable conversations between a therapist and a patient that follow their own rules of discursive structuring, while the second dimension occurs in the dialogical exchange that arises between the multiple voices of an individual from their different positions adopted in the course of psychotherapy (Martínez et al., 2012, 2014).

A study that analyzed change episodes and rupture alliance episodes from five psychotherapies of different orientations identified a higher probability of patients interacting from the reflective subjective position in the former, and that this probability increases as the episode progresses, while the probability of interacting from the independent subjective position decreases. In therapists, no differences were observed in the interaction between the proposer and professor subjective positions in the two types of episodes (Morán et al., 2016).

Dyadic interactions between therapists and patients can be understood as a self-organized system with emerging properties, a dynamic organization that manifests itself in a nonlinear way over time (Tschacher and Haken, 2007; Salvatore and Tschacher, 2012; Schiepek et al., 2014; Tschacher et al., 2015). This dynamic organization can go through critical moments between different significant moments of psychotherapy, those that can precede the formation of novel patterns that grant new stability to the system and are, at the same time, a manifestation of therapeutic change (Schiepek et al., 2014). Considering a dialogical point of view of the change in psychotherapy, reorganization of the subjectivity (Martínez and Tomicic, 2019) of patients throughout the process could be considered as an emerging quality that is established from interactions between patients and therapists, a self-organized system. The interaction between voices from different personal positions of patients and therapists can form patterns over time, i.e., dynamic patterns, which can present periods of stability that, combined with periods of instability, can have important consequences for patient change.

The inner dynamics of systems and their conditions of instability can cause variations in their parameters or characteristic tendencies, generating emerging qualities (Prigogine, 2004) that can be considered as novel behaviors of the system. Dynamical Systems (DS) can manifest minimal, gradual, and continuous changes (first-order changes) while maintaining dominant patterns of functioning, called attractors, and discontinuous and abrupt changes each time their dominant patterns are affected by critical instabilities and turbulence, called repellors, allowing them to reorganize their behaviors into qualitative novel patterns (second-order changes) (Prigogine and Stengers, 1994). Systems far from equilibrium tend to self-organize by constructing feedback cycles between their subsystems, forming their structures (Guastello and Gregson, 2011) through information flow patterns (Haken, 2010). Moreover, emerging qualities can be understood as recursive and stable patterns in time that arise from the dynamic relationship of the elements of a system without a defined precondition. An attractor is a state of DS in which its constituent elements are organized into preferred and relatively stable patterns, to which the system returns whenever it is perturbed (Hayes and Yasinski, 2015). In terms of behaviors, they remain stable until a sufficiently significant threshold is reached, which moves the system toward other potential states. The behavior of the elements of a system is defined by a multitude of attractors, which build a dynamic landscape that constrains the possible future behavior of a system, defining behaviors of higher and lower probabilities (Quinlan, 2017).

In this research field, attractors have been identified in relevant moments of psychotherapy associated with therapeutic change, in innovative moments of a patient at the end of treatment, in the interaction between discourse and vocal quality patterns of patients and therapists, and in activation of a positive network (which promotes change) that seems to remain stable in depressive patients in the final phase of their treatment (Ribeiro et al., 2011; Hayes et al., 2015; Tomicic et al., 2015b), suggesting that it is not only possible to recognize these properties of systems in psychotherapy, but that it is possible to do so through the discourse and narratives of patients and therapists during psychotherapy.

Observing a change in the subjectivity of a patient in the context of their therapeutic relationship can be valuable in a clinical case where, according to the patient’s diagnosis, a process that starts from an experience of dissociation (or vulnerability of the self) can be integrated by psychotherapy.

Based on this suggestion, at least four aspects could form a vulnerable state in the self of patients diagnosed with BPD, opening the possibility of finding out rigid and disperse self-states: (a) considering affective instability, greater lability in terms of anger and anxiety, and oscillation between depression and anxiety but not in terms of oscillation between depression and elation (Koenigsberg et al., 2002); (b) some studies have pointed out that patients diagnosed with BPD show a deficit in the regulation of emotions, insecure attachment styles, ultra-reading, and incorrect reading of the mind, which can be considered predictors of the traits of the patients (Ghiasi et al., 2016); (c) early traumatic experiences, emotional abuse, and neglect in childhood are widely present in the development of BPD (Bierer et al., 2003; Zhang et al., 2012; Stevenson et al., 2016), and while physical and sexual abuse has been associated with an externally directed (i.e., paranoid and antisocial) reaction, emotional abuse has been associated with affective instability, impulsivity, and self-directed aggressive behaviors (Bierer et al., 2003); (d) the presence of levels of dissociation that can affect identity and identity diffusion problems (Scalabrini et al., 2017; Luyten et al., 2020). In BPD, dissociative experiences may be associated with disturbances occurring in the environment and difficulty in sustaining adaptive cognitive and emotional functioning (Laddis et al., 2017).

Thus, a case study was designed to answer the following research questions: what dynamic patterns emerge in the personal positions of a patient diagnosed with BPD and her therapist during change episodes of long-term psychotherapy? How are the dynamic patterns associated with the subjective change process of the patient?

The hypotheses were as follows: (1) dynamic patterns will be found in personal positions of the patient and her therapist during change episodes in the psychotherapy and (2) differentiated dynamic patterns will be found in personal positions of the patient and her therapist in change episodes depending on the level of subjective elaboration of the episodes.

## Materials and Methods

A mixed methodology was developed in a single case study which considered the search of dynamic patterns of personal positions of the patient and her therapist in relevant change episodes identified in long-term psychotherapy. Change episodes were determined through the Change Episodes Model (Krause et al., 2006, 2015), which is a procedure that has been applied in different types of psychotherapies and patients with varied diagnoses (e.g., Krause et al., 2007; Tomicic et al., 2015a; Mellado et al., 2017) to evaluate the change process of patients in a generic way that is covering any model of psychotherapy regardless of its theoretical background. In these episodes, the voices and personal positions of an adult patient diagnosed with BPD and her therapist were obtained by qualitative analysis following the Model of Analysis of Discursive Positioning in Psychotherapy (MAPP) (Martínez and Tomicic, 2019), which is a model based on the dialogical self theory that considers both patient and therapist a basic element to be able to define dynamic pattern formation in the manifestations of their subjective states. Later, a quantitative analysis was conducted, by which patterns in the voices and personal positions of the patient and her therapist were identified, describing hypothetical attractors according to the specific level of subjective change achieved by the patient in the change episodes. These attractors were evaluated using the Space State Grid (SSG) technique, designed from a conceptualization of the DS perspective in psychology (Lewis et al., 1999; Hollenstein, 2013).

### Participants

The participants were a female adult outpatient (31 years old) diagnosed with BPD by a treating psychiatrist who made her referral to psychotherapy, and her therapist, an adult man (53 years old). The patient received psychodynamic treatment (44 sessions) in a public psychiatric institute in Santiago de Chile. The therapist was a psychologist with 25 years of experience as a psychodynamic therapist. The reason for the consultation of the patient was severely aggressive behavior toward her 6-year-old daughter. The patient was married, mother of two school-aged children. The patient sometimes beat up her daughter, but she was able to recognize the harm she was causing to her daughter, so she stopped doing it even though she sometimes grabs her daughter and shouts violently at her. This lack of recognition also happens when she drinks alcohol on weekends, turning her into “someone else”: someone uninhibited, daring, and sensual. In addition, she describes herself as a shy, bashful, and foolish woman.

She grew up in an environment that bestowed upon her the importance of cleanliness, order, and good habits, but that at the same time harmed her through physical and psychological abuse. She was sexually abused twice when she was aged between 8 and 9 years of age by friends of the family. The mother of the patient committed suicide 5 years before she sought help, while her father died (because of cirrhosis) during psychotherapy.

Considering the results of the OQ-45.2 (Lambert et al., 1996; von Bergen and de la Parra, 2002) applied to the patient, the therapy was considered a successful treatment. In the final session, her score was 51 points, with 73 points being the cut-off point for the clinical population in Chile. Her initial score was 123 points; therefore the Reliable Change Index (RCI) was higher than seventeen points, the expected difference between the final and first sessions.

### Procedure

The patient and the therapist were invited to participate in the context of the research project “Mentalizing in psychotherapeutic processes with patients diagnosed with a personality disorder: Its role in mutual regulation and its association with therapeutic change” (funded by FONDECYT, project number 1150639). The ethical protocol for this study was approved by the Scientific Ethics Board of Universidad Diego Portales, and informed consent forms were signed by both the patient and the therapist, who allowed the therapy material to be used for research purposes and related publications. In addition, the ethical process was approved by the Scientific Ethical Committee of Social Sciences, Arts and Humanities, of the Pontificia Universidad Católica de Chile, ID: 181108008.

To ensure the quality and reliability of the analyses, the data were developed by conciliation utilizing interpreter triangulation (Patton, 2015) based on an inter-subjective reliability process (Flick, 2015) while also ensuring rigorous analysis with the assistance of the Atlas-Ti 7.5 software (Friese, 2013). All sessions of the psychotherapy were video-recorded and transcribed following the guidelines proposed by Mergenthaler and Gril (1996).

#### Change Episode Identification

A qualitative stage was realized where change moments of the patient in psychotherapy were identified through the Change Episodes Model (Krause et al., 2006; Krause et al., 2007; Krause et al., 2015). Change episodes are segments that contain moments of patient change during a psychotherapy process and, as such, correspond to relevant episodes of psychotherapy that allow for understanding of the connection among aspects of the psychotherapeutic process and its results. From this approach, psychotherapeutic change is, fundamentally, the creation of a subjective theory. The whole change episodes comprise change moments of the patient at the end of the episode, and the previous interaction between the patient and the therapist related to the thematic content of the change moment.

The method for determining change episodes is based on the notion of therapeutic change as a transformation of patients’ subjective theories about themselves, their relationship to the environment, and their problems (Krause et al., 2007). Subjective change is operationalized through the “Generic Change Indicators” (GCIs) (refer to Table 1; Krause et al., 2006), which identify the contents of change (Krause et al., 2007). The delimitation of change episode extends from the moment in which the therapist and the patient began to talk about a specific content of the change and ends with a moment of change (Krause et al., 2006).

**TABLE 1:** Generic change indicators (taken from Krause et al., 2015).

Change level	Generic change indicators
I. Initial consolidation of the structure of the therapeutic relationship	1. Acceptance of the existence of a problem 2. Acceptance of his/her limits and of the need for help 3. Acceptance of the therapist as a competent professional 4. Expression of hope 5. Questioning of habitual understanding, behavior and emotions 6. Expression of the need for change 7. Recognition of his/her own participation in the problems
II. Increase in permeability toward new understandings	8. Discovery of new aspects of self 9. Manifestation of new behavior or emotions 10. Appearance of feelings of competence 11. Establishment of new connections (self, surrounding, and own biography) 12. Reconceptualization of problems and/or symptoms 13. Transformation of valorizations and emotions in relation to self or others
III. Construction and consolidation of a new understanding	14. Creation of subjective constructs of self through the interconnection of personal aspects and aspects of the surroundings, including problems and symptoms 15. Founding of the subjective constructs in own biography 16. Autonomous comprehension and use of the context of psychological meaning 17. Acknowledgment of received help 18. Decreased asymmetry between patient and therapist 19. Construction of a biographically grounded subjective theory of self and of his/her relationship with surroundings

Change episodes can be classified into three hierarchical levels according to the GCI assigned to the moment of change presented by the patient (Altimir et al., 2010). The change episodes of level I correspond to early changes in the patient that contribute to the initial consolidation of the structure of the therapeutic relationship (GCI 1–7), i.e., “Recognition of the existence of a problem” (GCI 1), “Acceptance of the therapist as a competent professional” (GCI 3), or “Recognition of his/her participation in the problems” (GCI 7). Change episodes of level II correspond to changes that account for increase in permeability to new understanding (GCI 8–13), i.e., “Discovery of new aspects of self” (GCI 8) or “Reconceptualization of problems and/or symptoms” (GCI 12). Finally, Change episodes of level III correspond to changes that express the construction and consolidation of new understanding (GCI 14–19), i.e., “Creation of subjective constructs of self through the interconnection of personal aspects and aspects of the surroundings, including problems and symptoms” (GCI 14) or “Autonomous comprehension and use of the context of psychological meaning” (GCI 16).

Two coders trained in the observation of change episodes (Krause et al., 2006, 2007) independently observed all the videotaped and transcribed sessions of the therapy, identified change moments in the patient, and suggested some coding corresponding to one of the 19 GCIs of the model. Later, with the active participation of a trained judge, it is confirmed that both coders have independently registered moments of change in the same period of the session or come to an inter-subjective agreement regarding the presence of a moment of change when one of the coders has registered it. Finally, the two coders and the judge delimit the change episodes according to their thematic correspondence and assign a number considering the appropriate indicator.

#### The Model of Analysis of Discursive Positioning in Psychotherapy

Voices and personal positions of the patient and therapist were identified in change episodes through the MAPP (Martínez and Tomicic, 2019).

The MAPP allows to recognize how the subjectivity of patients and therapists is organized in discourse, depending on the interactive context of which it forms part. This implies that such subjectivities acquire form and meaning according to social and cultural rules that govern the practice of psychotherapy (Martínez et al., 2014). This model points out that a personal position is equivalent to a subjective position (or self-state), that is, a point of view or way of being in the world that is expressed in statements that form part of a subject’s discourse. Thus, a statement may contain more than one point of view or position, which constitutes its multivoiced aspect. While these positions are implicit in discourse, they are likely to be made explicit through linguistic markers (Martínez et al., 2012).

The MAPP can be considered a three-level system (Martínez and Tomicic, 2019). The first level contains voices of an individual, which constitute his/her idiosyncratic expression of subjectivity in the discourse. The voices can be related to each other by the definition that patients and therapists make of themselves and their environment, and are grouped in certain personal positions. The second level consists of the idiosyncratic positions of individuals and groups repertoires of voices that represent or vocalize them. Finally, the third level corresponds to an abstract taxonomy that represents the typical organization of idiosyncratic positions of individuals. The repertoire of voices can be diverse and unique for each patient, since it represents very specifically how their subjectivity is organized, while for therapists it can be less flexible since their voices express their point of view in a professional role. Although the first level is theoretically idiosyncratic, the second level presents personal positions that account for a specific repertoire of voices involved in a common subjectivity, which is part of the multiple states of self that can be adopted by patients and therapists in psychotherapy; therefore, common aspects can be found that allow us to establish similar personal positions among individuals. Regarding the third level, which presents an abstract taxonomy of the configurations of the positions adopted by patients and therapists in psychotherapy, the analysis of different psychotherapies has, so far, led to the identification of three typical configurations in patients, reflective, dependent, and independent, and two for therapists, proposer and professor (see Table 2).

**TABLE 2:** Personal positions according to Model of Analysis of Discursive Positioning in Psychotherapy (MAPP) taxonomy (taken from Martínez and Tomicic, 2019).

Personal positions of the patients	Description
The reflective	The reflective general category accounts for a subjective state in which the patient can have a distant – but not disconnected – perspective of emotional situations, listening to and critically looking at other aspects of the self while encouraging dialogue between them.
The dependent	The dependent general category is characterized by the self-positioning of patients as needy, weak, damaged, and/or vulnerable.
The independent	The independent general category is characterized by the positioning of the patient as someone strong and self-sufficient and/or as someone who does not need help from others.
**Personal positions of the therapists**	**Description**
The proposer	The proposer, in which the therapist positions him/herself as someone who shows the patient what he observes and offers him a new perspective, thus trying to generate a space for dialogue between the patient’s positions.
The professor	The professor is more dominant and monological, establishing the therapist as someone in possession of truth or knowledge that can be presented, taught, or sometimes imposed on the patient as the only alternative.

Once the change episodes were identified along the course of all videotaped sessions; it was necessary to observe the first three sessions and to code the segments of the discourse that were considered relevant before applying the MAPP system to such episodes. In this way, the following steps were accomplished:

##### Step 1: Identification of Voices

The first three sessions of therapy were coded to identify recurrent enunciators in the discourse of the participants: the therapist and the patient. An enunciator is a point of view expressed in statements by means of voice. An enunciator not only expresses spoken contents but also the perspective from which that content is mentioned. To do this, it is necessary to read each speech turn and code it to answer the following question: what are the points of view or perspectives expressed in what is being said? This makes it possible to identify recurrent enunciators in the speech of the patient and the therapist, which will make it possible to determine the voices present in speech turns (Martínez and Tomicic, 2019).

Afterward, the change episodes of therapy were analyzed, looking for the discursive voices found to characterize, refine, and improve saturation. It is necessary to accurately identify the markers of each identified discursive voice. It seeks to answer the following question: “How do discursive voices typically speak?”

##### Step 2: Determination of Idiosyncratic Personal Positions

At this stage, the set of discursive voices was categorized and named as an idiosyncratic personal position, that is, belonging to a particular patient or therapist. To do this, the discursive voices of each participant, the patient and the therapist, were grouped into more abstract and inclusive categories. Each category was labeled according to the common subjectivity that involves each specific set or repertoire of discursive voices. The purpose was to answer the question “From what perspective does each of these voices speak?” (Martínez and Tomicic, 2019).

##### Step 3: Classification of Idiosyncratic Personal Positions in the General Taxonomy of the Model of Analysis of Discursive Positioning in Psychotherapy

The general taxonomy of the MAPP represents the configuration of personal positions that are typically adopted by patients and therapists in psychotherapy. This taxonomy has emerged from the application of the two previous stages to different psychotherapies and categorization of idiosyncratic personal positions for both patients and therapists in different treatment modalities. Once steps 1 and 2 have been completed, classification of different idiosyncratic personal positions in the corresponding abstract categories is carried out.

To apply the MAPP, the step 1 described above was followed by grouping the identified voices into suggested personal positions that were then contrasted with those described in the MAPP taxonomy through a process based on Consensual Qualitative Research (Hill et al., 1997, 2005). Two independent coders observed the transcriptions of the first three sessions identifying specific contents and markers about how patient and therapist express relevant aspects of their subjectivity in the therapeutic conversation (domain and core ideas). A preliminary definition that allowed to guide the later categorization of idiosyncratic personal positions and respective voices was assigned to each of the general domains. Finally, each domain was discussed and agreed upon, with the active participation of the judge, and then categorized into voices; the latter were grouped in different personal positions (cross-analysis). These data were contrasted in the general taxonomy of the MAPP to evaluate their fit with the personal positions that already emerged from previous research on psychotherapy. An internal researcher (member of the team) served as a judge in each of the stages of data construction (auditing). At this stage, the judge assumed a critical role in safeguarding the fidelity of the general contents and their definitions in the subjective expression that was manifesting in psychotherapy. Subsequently, steps 2 and 3 were performed according to the same criteria on each speech turn of each identified episode. According to one of the inter-coder agreement measures provided by Atlas.ti 7.5, the two coders, before reaching an inter-subjective agreement, obtained a Holsti Index of 75.7%.

The description and characterization of the personal positions and voices of this therapy can be found with the following link, https://osf.io/mceak/.

#### Identification of Dynamic Patterns Between Therapists and Patients in Change Episodes of the Psychotherapy

The SSG is a technique developed by Lewis et al. (1999) and adapted by Hollenstein (2013); it consists of a graphical approach that allows for the use of categorical data to quantify them according to two space dimensions (the *x* and *y* axes) that define the state (the space of all possible states) of a given system. Any time series of two or more synchronized streams of categorical data can be used as inputs. In general, the SSG technique can be useful in three ways: (1) a visual tool to represent time patterns between two or more variables synchronized in time, (2) an exploratory tool to develop hypotheses on processes that unfold over time, and (3) a source of measurements not available with existing methods. Each SSG and the measures derived from it can depict a single path, a selected group of paths, and/or a complete sample (Hollestein, 2013).

To account for the association of voices and personal positions in the patient and her therapist, the data of the voices and personal positions that were obtained from each change episode were combined at the level of the speech turns. Once these data were constructed, they were imported into the Grid Ware Software (Lamey et al., 2004) for analysis considering the voices and personal positions of the patient and her therapist during change episodes. The voices and personal positions present in all change episodes (and their respective three levels) were considered, giving an account of hypothetical attractors that could be established in this process. In each cell of each grid, the *x*-axis depicts the categories of patients’ voices and personal positions, while the *y*-axis represents the categories of therapists’ voices and personal positions. The dots in the grid depicted the number of events, that is, each time the patient and the therapist remained in a definite state during their speech turns during the change episodes. The latter means that a node was depicted each time the patient spoke with a certain voice and the therapist spoke with a certain voice in two consecutive speech turns.

For the identification of hypothetical attractors, the winnowing technique (Lewis et al., 1999; Hollenstein, 2013) was utilized. To apply this technique, the mean visits [according to Lamey et al. (2004) a cell visit is one or more consecutive events occurring in a single cell, beginning upon a trajectory’s entry into the cell and ending upon its exit] were used of the voices and personal positions of the patient and the therapist in change episodes. The procedure began by ordering all cells that had at least one event and progressed iteratively, eliminating cells with the lowest average in each step (in total between seven and 27 iteration steps were performed). In each step, a heterogeneity score was calculated (refer to Equation 1) based on the observed and expected values for each cell. The null hypothesis was that all behaviors in the state space were equally distributed (the homogeneity was high) such that each cell’s mean visits were the same. To identify which cells constitute a hypothetical attractor, the heterogeneity values were quantified as a proportion of heterogeneity, taking as reference the first value where all the cells are included. A value of 1 meant that all interactions between voices and personal positions included were equally distributed. The iteration process made it possible to find, depending on the case, when there was a significant drop in the proportion of heterogeneity [which is defined by Lewis et al. (1999) with a value of 0.5], thus accepting that the cells contained in the iteration were different from the remaining cells and could establish the identification of a hypothetical attractor.

Heterogeneity score from the winnowing method (Lewis et al., 1999),


(1) Heterogeneity=jΣ⁢(Observedi-Expectedj)2/Expectedj#⁢of⁢Cellsj

where *i* is an index of the cell and *j* is an index of the current iteration.

## Results

### Change Episodes During Psychotherapy

**FIGURE 1:**
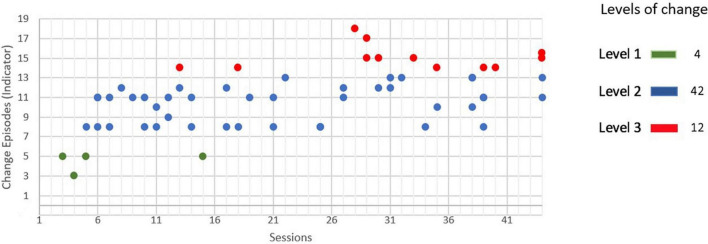
Change episodes of the patient in the psychotherapy (44 sessions).

The patient presented 58 change episodes during the psychotherapy. Of this total, 4 corresponded to level 1 (initial consolidation of the structure of the therapeutic relationship), 42 corresponded to level 2 (increase in permeability toward new understandings), and the remaining 12 episodes corresponded to level 3 (construction and consolidation of a new understanding). The distribution of the episodes in the different sessions according to the level of the indicator can be seen in Figure 1.

### Voices and Personal Positions of the Patient and Her Therapist in the Psychotherapy

In the patient, three idiosyncratic personal positions were identified: (1) the *integrative*, (2) the *incapable*, and (3) the *detached*. The *integrative position* is expressed through three voices: continuity, self-dialogue, and grounded voices. At the same time, according to the taxonomy of the MAPP, this position corresponds to the reflective position. The *incapable position* is expressed through a variety of voices: good-for-nothing, envious girl, fearful, confused and, sad and guilty voices, and according to the taxonomy of the MAPP, this position corresponds to the dependent personal position. The *detached position* is expressed through four voices: a voice of duty, and disaffectionate, angry, and carefree voices; according to the taxonomy of the MAPP, this position corresponds to the independent personal position. In the therapist, two idiosyncratic personal positions were identified: (1) the *proposer* and (2) the *expert*. The *proposer position* is expressed through four voices: inquirer voice, confrontational voice, meta-analytical voice, and self-revealing voice. In turn, according to the taxonomy of the MAPP, this idiosyncratic position corresponds to the proposer personal position. Finally, the *expert position* is expressed through two voices: the specialist’s voice and the asserting voice. In turn, according to the taxonomy of the MAPP, this idiosyncratic position corresponds to the personal position called professor. An outline of the personal positions, their respective voices, and the correspondence with the taxonomy of the MAPP can be seen in Table 3.

**TABLE 3:** Patient’s and her therapist’s voices, personal positions, and MAPP taxonomy.

Therapy	Patient’s personal positions and voices	Therapist’s personal positions and voices	MAPP taxonomy
Female patient and her therapist	**1. The integrative** 1.1. Continuity voice 1.2. Self-dialogue voice 1.3. Grounded voice	**1. The proposer** 1.1. Inquirer voice 1.2. Confrontational voice 1.3. Meta-analytical voice 1.4. Self-revealing voice	Patient 1. The reflective 2. The dependent 3. The independent
	**2. The incapable** 2.1. Good for nothing voice 2.2. Envious girl voice 2.3. Fearful voice 2.4. Confused voice 2.5. Sad and guilty voice	**2. The expert** 2.6. Asserting voice 2.7. Specialist’s voice	Therapist 1. The proposer 2. The professor
	**3. The detached** 3.1. The voice of duty 3.2. Disaffectionate voice 3.3. Angry voice 3.4. Carefree voice		

Below is an excerpt from the patient’s 5th change episode and its coding according to the MAPP. This episode was identified with GCI number 15, “founding of the subjective constructs in own biography” (level 3). The voices of both the therapist and the patient and their idiosyncratic personal positions are appreciated (see Table 4).

**TABLE 4:** Excerpt of change episode number 55 (level 3) from session 44 of the patient and their codification according to MAPP (T, therapist; P, patient).

Speak turns of therapist and patient	Codification according to MAPP
313. T: And that’s changed with your children?	Inquirer voice (proposer personal position)
314. P: Yes…now I’m more of an adult, now I’m already, now I’m talking to her…before it was like I gave her food…“ehh mom” she told me something about school and things like that, I…“ahh ok, yes, yes” I didn’t pay attention to her…	Grounded voice (integrative personal position)
315. T: mmm…(nodding)	
316. P: It was like…she was there, I had her because I had to have her…and now I don’t…	Self-dialogue voice (integrative personal position)
317. T: mmm…(nodding)	
318. P: Now we talk, now I say to her…she talks to me and I pay attention to her because before she talked to me and I did something else “ahh ok, good,” “bye, later I’ll come back” or so…	Grounded voice (Integrative personal position)

### Dynamic Patterns of Personal Positions in Change Episodes

Including all the change episodes the patient had in the psychotherapy and considering each of the three levels of subjective elaboration of the change, according to the grouping of change episodes, the following results in the personal positions were obtained.

**FIGURE 2:**
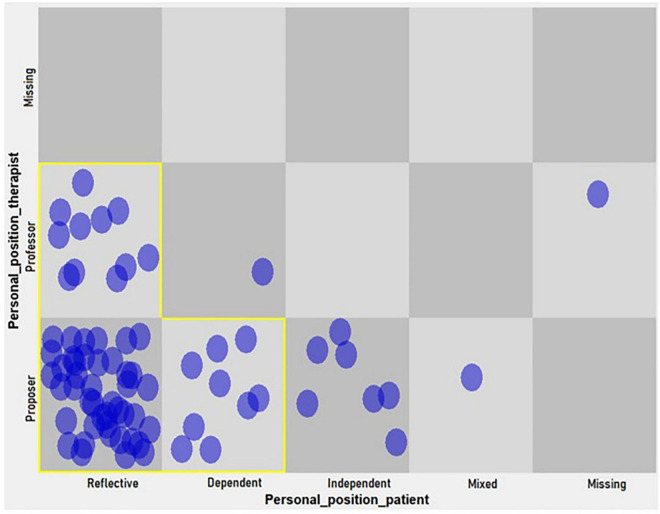
Dynamic pattern in personal positions of the patient and her therapist (level 1, change episodes).

(a) A dynamic pattern (hypothetical attractor) in level 1 change episodes (initial consolidation of the structure of the therapeutic relationship) emerged between the professor position of the therapist and the reflective position of the patient. Also, there was a pattern between the proposer position of the therapist and the reflective and dependent positions of the patient (Figure 2).

As shown in Table 5, the drop of more than 50% in the heterogeneity proportion marks the last three cells as hypothetical attractors.

**TABLE 5:** Derivation of attractors with heterogeneity score in a seven-used-cell (3 × 5) grid in level 1 change episodes.

Step	Visits (mean visits)										
	Missing	Mixed	Professor/Dependent	Proposer/Independent	Proposer/Dependent	Professor/Reflective	Proposer/Reflective	Total (V)	#Cells (C)	Expected (V/C)	
1	1	1	1	2	4	6	10	25	7	3.6	
2		1	1	2	4	6	10	24	6	4	
3			1	2	4	6	10	23	5	4.6	
4				2	4	6	10	22	4	5.5	
5					4	6	10	20	3	6.7	
6						6	10	16	2	8	
7							10	10	1	10	

	**(Observed − Expected)^2^/Expected**							**Sum**	**Cells**	**H-score**	**H-proportion**

1	1.9	1.9	1.9	0.7	0.1	1.7	11.6	20	7	2.79	100%
2		2.3	2.3	1	0.0	1	9.0	16	6	2.58	93%
3			2.8	1.5	0.1	0.4	6.3	11	5	2.23	80%
4				2.2	0.4	0	3.7	6	4	1.59	57%
5					1.1	0.1	1.7	3	3	0.93	33%
6						0.5	0.5	1	2	0.50	18%
7							0.0	0	1	0.00	0%

*Cells “professor/reflective,” “proposer/reflective,” and “proposer/dependent” are identified as a hypothetical attractor.*

**FIGURE 3:**
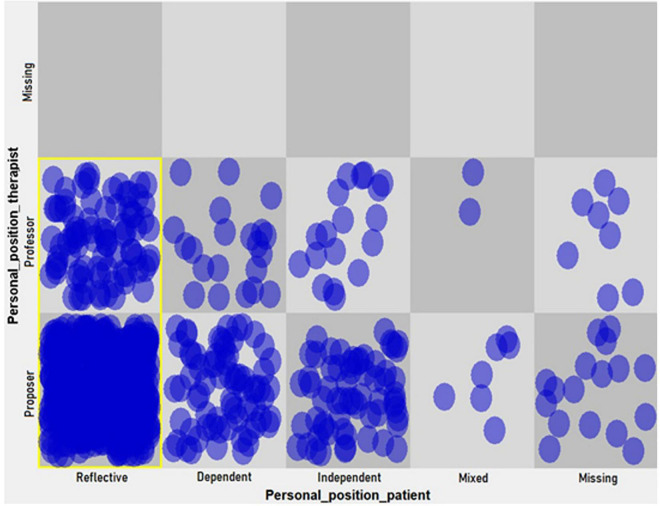
Dynamic pattern in personal positions of the patient and her therapist (level 2, change episodes).

(b) A dynamic pattern (hypothetical attractor) in level 2 change episodes (increase in permeability toward new understanding) emerged between the professor position of the therapist and the reflective position of the patient. Also, a pattern could be found between the proposer position of the therapist and the reflective position of the patient (Figure 3).

As shown in Table 6, the drop of more than 50% in the heterogeneity proportion marks the last two cells as hypothetical attractors.

**TABLE 6:** Derivation of attractors with heterogeneity score in a ten-used-cell (3 × 5) grid in level 2 change episodes.

Step	Visits (mean visits)													
	Mixed	Mixed	Missing	Missing	Professor/Independent	Professor/Dependent	Proposer/Independent	Proposer/Dependent	Professor/Reflective	Proposer/Reflective	Total (V)	#Cells (C)	Expected (V/C)	
1	2	4	6	9	10	12	20	24	32	73	192	10	19	
2		4	6	9	10	12	20	24	32	73	190	9	21	
3			6	9	10	12	20	24	32	73	186	8	23	
4				9	10	12	20	24	32	73	180	7	26	
5					10	12	20	24	32	73	171	6	28	
6						12	20	24	32	73	161	5	32	
7							20	24	32	73	149	4	37	
8								24	32	73	129	3	43	
9									32	73	105	2	53	
10										73	73	1	73	

	**(Observed − Expected)^2^/Expected**										**Sum**	**Cells**	**H-score**	**H-proportion**

1	15.4	12	9.1	5.4	4.4	2.7	0	1.2	8.5	150.8	209.5	10	20.95	100%
2		13.9	10.8	7	5.8	3.9	0.1	0.4	5.6	127.6	175	9	19.45	93%
3			12.8	8.7	7.6	5.4	0.5	0	3.3	106.5	145	8	18.09	86%
4				11	9.6	7.3	1.3	0.1	1.5	87	118	7	16.81	80%
5					12.0	9.5	2.5	0.7	0.4	69.5	95	6	15.79	75%
6						12.7	4.6	2.1	0	51.7	71	5	14.22	68%
7							8.0	4.7	0.7	34.3	48	4	11.94	57%
8								8.4	2.8	20.9	32	3	10.72	51%
9									8.0	8	16	2	8.00	38%
10										0	0	1	0.00	0%

*Cells “professor/reflective” and “proposer/reflective,” are identified as a hypothetical attractor.*

**FIGURE 4:**
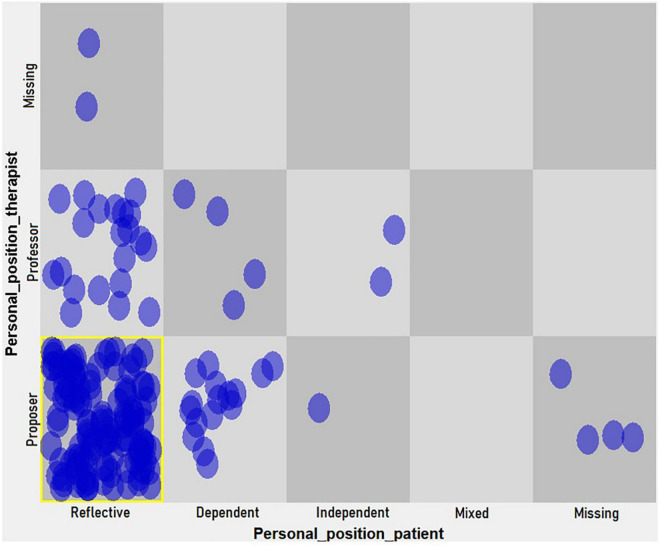
Dynamic pattern in personal positions of the patient and her therapist (level 3, change episodes).

(c) A dynamic pattern (hypothetical attractor) in level 3 change episodes (construction and consolidation of a new understanding) of the therapy emerged between the proposer position of the therapist and the reflective position of the patient (Figure 4).

As shown in Table 7, the drop of more than 50% in the heterogeneity proportion marks the last cell as a hypothetical attractor.

**TABLE 7:** Derivation of attractors with heterogeneity score in an eight-used-cell (3 × 5) grid in level 3 change episodes.

Step	Visits (mean visits)											
	Proposer/Independent	Missing	Professor/Independent	Professor/Dependent	Missing	Proposer/Dependent	Professor/Reflective	Proposer/Reflective	Total (V)	#Cells (C)	Expected (V/C)	
1	1	1	1	2	4	5	5	18	37	8	4.6	
2		1	1	2	4	5	5	18	36	7	5.1	
3			1	2	4	5	5	18	35	6	5.8	
4				2	4	5	5	18	34	5	6.8	
5					4	5	5	18	32	4	8	
6						5	5	18	28	3	9.3	
7							5	18	23	2	11.5	
8								18	18	1	18	

	**(Observed − Expected)^2^/Expected**								**Sum**	**Cells**	**H-score**	**H-proportion**

1	2.8	2.8	2.8	1.5	0.1	0	0	38.7	48.9	8	6.11	100%
2		3.3	3.3	1.9	0.3	0	0	32.1	41	7	5.86	96%
3			4	2.5	0.6	0.1	0.1	25.4	33	6	5.45	89%
4				3.4	1.2	0.5	0.5	18.4	24	5	4.79	78%
5					2	1.1	1.1	12.5	17	4	4.19	69%
6						2	2	8	12	3	4.02	66%
7							3.7	3.7	7	2	3.67	60%
8								0	0	1	0	0%

*Cell “proposer/reflective” is identified as a hypothetical attractor.*

### Dynamic Patterns of the Voices in the Change Episodes

To describe which voices particularly express the personal positions found in level 3 change episodes, a similar procedure was performed with the SSG technique.

**FIGURE 5:**
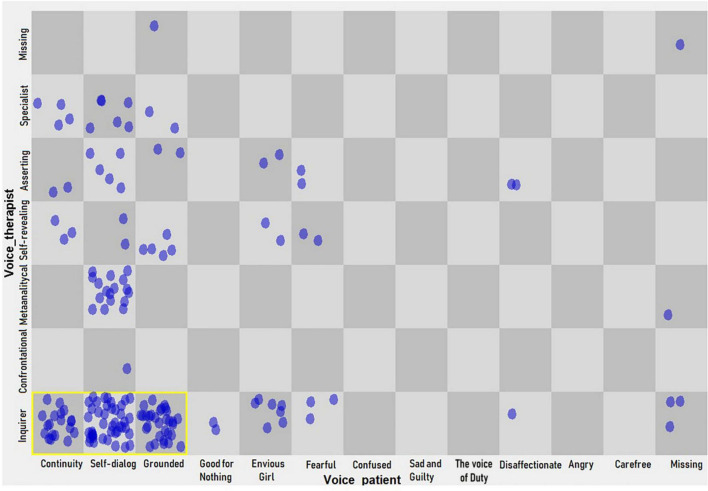
Dynamic pattern in voices of the patient and her therapist (level 3, change episodes).

(d) A dynamic pattern (hypothetical attractor) in the level 3 change episodes (construction and consolidation of a new understanding) of the therapy emerged between the therapist’s inquirer voice and the patient’s continuity, self-dialogue, and grounded voices (Figure 5).

## Discussion

The results of this case study indicate that during the patient’s change episodes, specific dynamic patterns of personal positions of both the patient and the therapist were identified depending on the phase of her subjective change (levels 1, 2, and 3 in GCI). The patterns changed as the degree of subjective elaboration was higher, starting with patterns that included an interplay between the therapist’s “professor” and “proposer” positions until arriving only at the “proposer” position in the episodes of greater subjective elaboration of the patient (level 3). Specifically, in this last group of change episodes, the voices that formed a dynamic pattern are the therapist’s “inquirer” voice, and the three voices that form part of the patient’s reflective position, that is, the “continuity,” “self-dialogue,” and “grounded” voices. During these higher-level change episodes, the therapeutic conversation of the patient and her therapist is being specified toward a pattern that can be described as an interaction between the “proposer” position of the therapist and the “reflective” position of the patient. While at levels of changes 1 and 2, other positions emerge, the proposer-reflective pattern remains stable throughout therapy, being the only pattern in later phases of change. The consecutive change that this patient had seems to be established from a dialogue in which the relevant topics presented by the patient were treated as life situations that can be thought of from her own experience, which in turn were integrated reflexively by the patient. In this way, a constructive process of association seems to be stimulated, which allows her to elaborate on new understanding.

These results are consistent with those found in previous studies (Tomicic et al., 2015b; Morán et al., 2016) regarding the patient’s achievement of a subjective positioning of greater openness to dialogue in change episodes, and the non-predominance of either of the two positions, professor and proposer, of the therapist, which were considered as organized speech from their therapeutic role independent of interactional contexts in which they found themselves. However, in this study, a difference was observed in the more elaborate episodes of change (level 3), in which the “proposer” position takes prominence, possibly suggesting specialization of its therapeutic role in a propositional aspect, possibly coherent with the much more reflective subjective manifestations of the patient. This aspect should be further researched in future studies involving other cases. The above studies have described pattern formation in psychotherapy as one of its features. These patterns seem to follow a non-linear distribution, as also reported for the formation of self-organized patterns in self-presentation categories of patients and therapists, wherein few patterns occur with a high frequency, which decreases following the inverse power law (IPL) distribution (Strunk and Lichtwarck-Aschoff, 2019), that is consistent with the identification of a few hypothetical attractors.

Considering the dynamic patterns of voices in level 3 of the change process, the “continuity,” “self-dialogue,” and “grounded” voices are expressions of the reflective position of the patient, suggesting that the process of creation and consolidation of new subjective constructs supports the building of an integrative psychological state. The integration of the three reflective voices can be understood as a dialogue of parts of the self that, at some point in the therapeutic process, may have been differentiated, as suggested by an assimilation process (Honos-Webb and Stiles, 1998; Stiles, 2011). Therefore, it would be important to evaluate if such a process was taking place between the voices of this patient, as well as if there were warded-off voices, dominant voices, and bridges of meaning between them. The voices’ interplay could be considered in the notion of “multiplicity in unity” (Hermans, 2008) that points to the result of dialogical relationships among different aspects of the self, overcoming fragmentation (from there the process of change experienced by the patient becomes relevant, from their “confused” and “fearful” voices that allude to diffusion of the experience from their self-definition, and that could be considered one aspect of the BPD diagnosis made in the psychiatric center where they had treatment) and arriving at the appearance of alternative positions or new relationships between these aspects of self. In this context, the “inquirer” voice of the therapist could be an offering to new ways of thinking about the content that the patient presents in therapy and the patient’s possibility of associating different contents from a position in which she establishes herself as the author (and the result) of what she is thinking.

Dissociation has been described as not a specific characteristic of patients diagnosed with BPD, being even heterogeneous and serving an avoidant function in the face of experiences that would involve excessive demand (Scalabrini et al., 2017). Although the dissociation of patients from the dialogical self theory basically refers to the disconnection of self states, it shares with other perspectives its protective function against fragmentation in response to traumatic experiences (Schimmenti and Caretti, 2016) and that the goal of a psychological treatment should assume the integration of mental and bodily states. This can be at the same time a starting point and an arrival point to evaluate the change process, which has been the central focus of this study. Future research in this area could confirm the subjective configurations that have emerged in the different change episodes, as they can serve as a guide to assess the psychotherapy progress with patients diagnosed with BPD or others, focusing on the evolution toward specific patterns and articulation of voices, especially within their reflective positions.

The patient’s changes have occurred in an interactional context in which the therapist had a role that can be considered from the changes in the patterns of his personal positions. In this sense, this relational notion can be complemented with other relevant theoretical-clinical aspects such as exploration of moments of connection between a patient and a therapist (i.e., looking for subtypes of the patients’ reflective voices and the therapists’ inquirer voices that can be protagonists in these moments) that help the patient’s process of subjective reorganization. Furthermore, this could also be supported by temporal-spatial realignment/integration of the self/others at neuronal and psychological levels (Scalabrini et al., 2020a,b), a process that could be explored from studies that also cover the salient characteristics of neurophysiological activities associated with patients’ change.

This case study points out that the verbal communication established in psychotherapy can trigger an opening to different aspects of the self, which previously could be feared, avoided, or pathologically maintained, or are part of the unconscious resources of patients. It is the idea of psychotherapy as a context where healthy aspects of the self help in the modification of more dysfunctional parts and give them a role in a broader and more adaptive setting (Hermans and Dimaggio, 2004). Communication, understood in this way and from the perspective of different positions and interacting voices, can help to understand these types of characteristics of change. It is a notion that underlies the content of therapeutic conversation.

Salvatore et al. (2008) consider therapeutic communication as a self-organized system. Although they describe some semantic components of the processes of meaning construction, they avoid external attributions in their interpretation. However, the MAPP applied here gives an account of subjective positions and voices from where diverse semantic components can be constructed. Both models seem to highlight the idiosyncratic character of the subjective elaboration of content in the therapeutic context. Salvatore et al. (2010) do not refer to the specific speech of patients and therapists under the assumption that the meaning-constructing process is an emerging property of verbal exchange. They emphasize that they are studying the process itself, something like what is achieved with the MAPP by describing personal positions in the therapeutic relationship during some relevant moments of psychotherapy. In addition, psychotherapy is characterized by nonlinearity and non-ergodicity. Other systemic studies have highlighted the importance of recognizing their idiographic aspects before generating models with aggregated data. For this, individual spectral analyses have been performed in a study on anxiety levels and therapeutic outcomes (Fisher et al., 2011) or network analyses to account for individual variability in the interaction between intersession processes (ISPs) and symptom stress of patients (Kaiser and Laireiter, 2018). The results found in this study can be considered as an idiographic step that will allow similarities to be recognized in other cases.

With regard to some therapeutic implications that can be obtained from this research, it is worth highlighting not only the description that has been made of the change in the patient’s subjectivity but also some consequences of identifying voices and personal positions during the therapeutic process. Thus, it may be significant to recognize the path that a BPD diagnosed patient makes toward the consolidation of a reflective position (“integrative” in this patient’s case), which is related to the proposer position of her therapist and something that was at the base of the elaborative work in her psychotherapy. In addition, some suggestions can be made regarding what a therapist can do with the information that emerges here. A therapist, in their professional training, can progress to self-recognize the voices and positions they usually use in psychotherapy and how they can be displayed depending on the patient being treated. The same can occur concerning the voices and positions of their patients, thus recognizing possible moments of change or enhancing therapeutic conversations from positions that are related to the change of the patient. Self-recognition and partial recognition of personal positions may be possible to the extent that already there are patterns identified that have been established at relevant moments in psychotherapy (e.g., change episodes and alliance rupture episodes). Some limitations of this case study are as follows: (1) as a case study, this research presents results that cannot be generalized; therefore, future studies could include change episodes and other types of episodes (that can also serve as a comparison group at change episode level) within other psychotherapies and recognize their dynamic patterns in the same way as here, observing if other aspects of clinical relevance can be recognized; (2) regarding the procedure of identification of hypothetical attractors, the duration of each interaction between voices of certain personal positions along the episode could be included, and/or other qualitative criteria could be added to refine its recognition in a specific state-space region (e.g., return time); (3) regarding the identification of personal positions, the MAPP seems an adequate instrument to describe clinically understandable subjective states, although it does not integrate other aspects of the dialogical self theory such as development of new voices arising from the influence of the therapist as “other” and operationalization of bridges of meaning that arise between voices, all of which are missing and need to be included in future research.

## Data Availability Statement

The data analyzed in this study is subject to the following licenses/restrictions: the data are anonymous and are safeguarded by the researcher responsible for the research. Requests to access these datasets should be directed to CM, claudio.martinez@mail.udp.cl.

## Ethics Statement

The studies involving human participants were reviewed and approved by the Comité de Ética en Investigación de la Universidad Diego Portales. The patients/participants provided their written informed consent to participate in this study. In addition, the ethical process was approved by the Scientific Ethical Committee of Social Sciences, Arts and Humanities, of the Pontificia Universidad Católica de Chile (ID: 181108008).

## Author Contributions

AM participated in the study design, data analyses, conclusion, and writing the draft of the manuscript. AT participated in the qualitative stage of data analysis. CM and MK participated in the study design and in the critical review of the draft of the manuscript. All authors contributed to the article and approved the submitted version.

## Conflict of Interest

The authors declare that the research was conducted in the absence of any commercial or financial relationships that could be construed as a potential conflict of interest.

## Publisher’s Note

All claims expressed in this article are solely those of the authors and do not necessarily represent those of their affiliated organizations, or those of the publisher, the editors and the reviewers. Any product that may be evaluated in this article, or claim that may be made by its manufacturer, is not guaranteed or endorsed by the publisher.
